# A resistant mutant of *Plasmodium falciparum* purine nucleoside phosphorylase uses wild-type neighbors to maintain parasite survival

**DOI:** 10.1016/j.jbc.2021.100342

**Published:** 2021-01-30

**Authors:** Yacoba V.T. Minnow, Rajesh K. Harijan, Vern L. Schramm

**Affiliations:** Department of Biochemistry, Albert Einstein College of Medicine, Bronx, New York, USA

**Keywords:** enzyme kinetics, drug resistance mechanisms, hybrid proteins, protein engineering, DADMe-ImmG, enzyme inhibitor, transition state analog, ACT, artemisinin-based combination therapy, DADMe-ImmG, 4′-Deaza-1′-Aza-2′-Deoxy-1′-(9-Methylene)-Immucillin-G, *Pfmdr1*, *P. falciparum* multidrug resistance gene 1, *Pf*Pgh1, *P. falciparum* P-glycoprotein homolog, *Pf*PNP, *Plasmodium falciparum* purine nucleoside phosphorylase, PPQ, piperaquine, TEV, tobacco etch virus, TSA, transition state analog

## Abstract

*Plasmodium falciparum* purine nucleoside phosphorylase (*Pf*PNP) catalyzes an essential step in purine salvage for parasite growth. 4′-Deaza-1′-Aza-2′-Deoxy-1′-(9-Methylene)-Immucillin-G (DADMe-ImmG) is a transition state analog inhibitor of this enzyme, and *P. falciparum* infections in an *Aotus* primate malaria model can be cleared by oral administration of DADMe-ImmG. *P. falciparum* cultured under increasing DADMe-ImmG drug pressure exhibited *Pf*PNP gene amplification, increased protein expression, and point mutations involved in DADMe-ImmG binding. However, the weak catalytic properties of the M183L resistance mutation (∼17,000-fold decrease in catalytic efficiency) are inconsistent with the essential function of *Pf*PNP. We hypothesized that M183L subunits may form mixed oligomers of native and mutant *Pf*PNP monomers to give hybrid hexameric enzymes with properties conferring DADMe-ImmG resistance. To test this hypothesis, we designed *Pf*PNP constructs that covalently linked native and the catalytically weak M183L mutant subunits. Engineered hybrid *Pf*PNP yielded trimer-of-dimer hexameric protein with alternating native and catalytically weak M183L subunits. This hybrid *Pf*PNP gave near-native *K*_m_ values for substrate, but the affinity for DADMe-ImmG and catalytic efficiency were both reduced approximately ninefold relative to a similar construct of native subunits. Contact between the relatively inactive M183L and native subunits is responsible for altered properties of the hybrid protein. Thus, gene amplification of *Pf*PNP provides adequate catalytic activity while resistance to DADMe-ImmG occurs in the hybrid oligomer to promote parasite survival. Coupled with the slow development of drug resistance, this resistance mechanism highlights the potential for DADMe-ImmG use in antimalarial combination therapies.

*Plasmodium falciparum* causes the most severe form of human malaria, a leading cause of mortality in Sub-Saharan Africa and Southeast Asia. In 2018, malaria accounted for 67% of the mortality in children under the age of five (1, 2). The World Health Organization estimated that in 2018, approximately 228 million people were infected with malaria, causing 405,000 deaths (1). *P. falciparum* is the predominant species in malaria endemic areas (3, 4).

The development of drug-resistant malaria parasites has plagued attempts at the eradication of malaria, as *P. falciparum* has developed resistance to nearly all approved antimalarial therapies. Emerging resistance to the current first-line therapy for *P. falciparum* malaria, Artemisinin-based Combination Therapy (ACT) in the Greater Mekong subregion, a region of high malaria endemicity, causes concerns of a new health crisis (5, 6, 7, 8). Developing resistance underscores the need for novel and targeted antimalarials to combat the emerging resistant parasites.

*P. falciparum* parasites are purine auxotrophs, and we have targeted purine metabolism as a potential drug target (9, 10, 11, 12). *P. falciparum* purine nucleoside phosphorylase (*Pf*PNP) is an essential enzyme in the purine salvage pathway, catalyzing the phosphorolytic breakdown of inosine to form hypoxanthine, the essential purine precursor (Fig. 1*A*; (9, 11, 13, 14, 15, 16)). Inhibition of host and *Pf*PNP with transition state analog inhibitors causes purine starvation and death of the parasite making *Pf*PNP an attractive drug target (13, 15, 17, 18).

**Figure 1:**
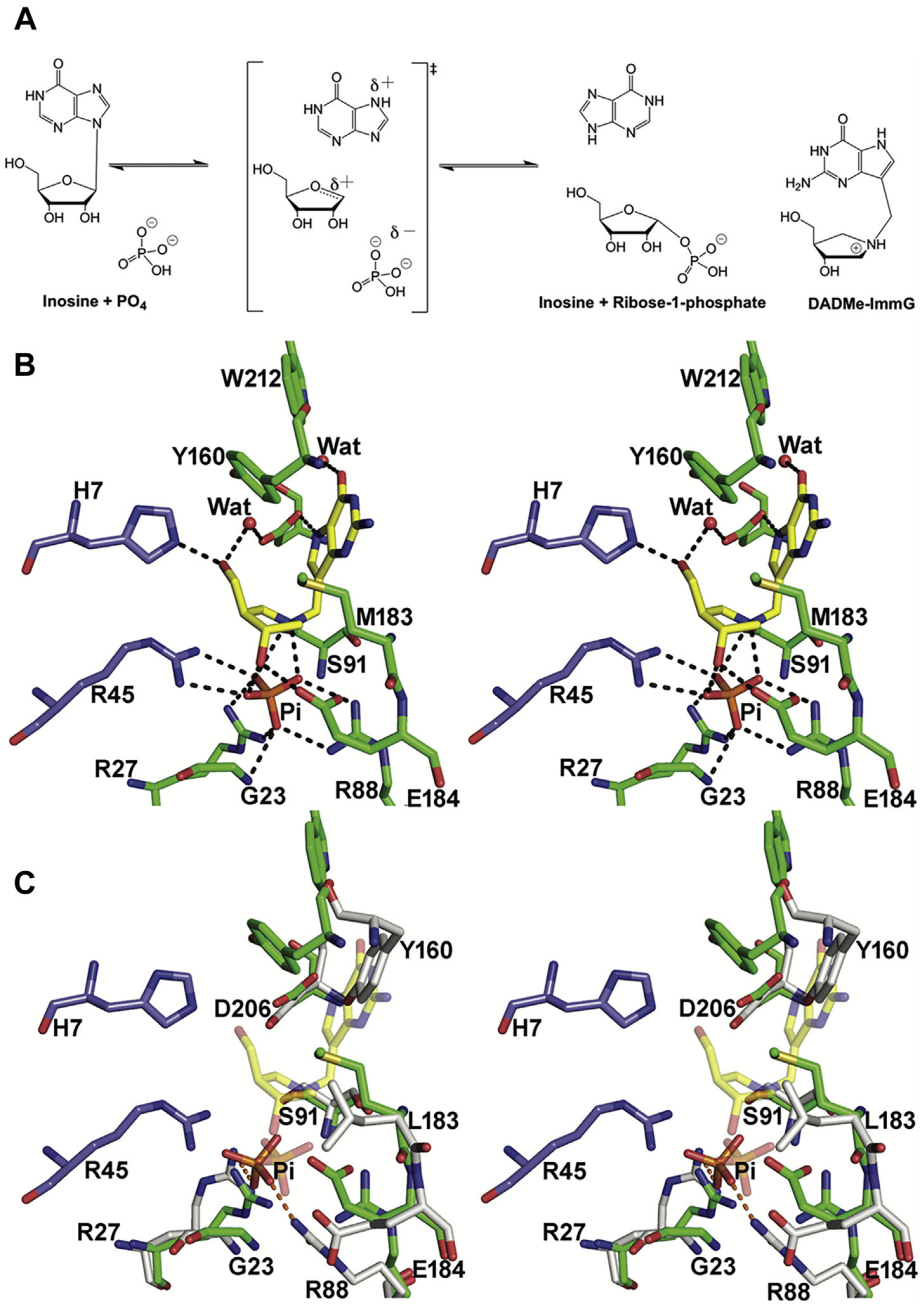
***A*, The phosphorolysis reaction catalyzed by *Pf*PNP. DADMe-ImmG is a transition state analog.***B*, stereoview of the catalytic site of *Pf*PNP-DADMe-ImmG-PO_4_ complex (PDB ID: 3PHC). Hydrogen bond interactions are indicated as *dotted lines*. Y160 and W212 form part of the hydrophobic pocket for adenine group binding. H7 and R45 are from the neighboring monomer. *C*, Active sites of native *Pf*PNP-DADMe-ImmG-PO_4_ (*green*, PDB ID: 3PHC), as in *B* are superimposed with M183L *Pf*PNP-PO_4_ (*gray*; PDB ID: 6AQU). M183L relocates the Y160 side chain into the catalytic site, perturbing catalysis and DADMe-ImmG binding (from 19).

DADMe-ImmG is a picomolar transition state analog (TSA) of PNP, which inhibits with a *K*_d_ of 670 pM and has an IC_50_ of 160 nM for parasites cultured in human erythrocytes (13, 19). The crystal structure of *Pf*PNP in complex with DADMe-ImmG (PDB ID: 3PHC) shows hydrogen bond and ion-pair interactions with active site residues, phosphate, and structural waters (Fig. 1*B*). These interactions define the tight-binding nature of DADMe-ImmG compared with the substrate inosine (PBD ID: 2BSX) and explain why DADMe-ImmG is such a strong inhibitor of *Pf*PNP. Resistance selection experiments indicated that PNP is the singular target for DADMe-ImmG. Resistance involves increased PNP protein expression and loss-of-function mutations occurring only in the PNP target enzyme (9, 19). Resistance to DADMe-ImmG develops slowly. After 1 year of drug pressure, modest resistance occurred as a consequence of threefold *Pf*PNP gene amplification. Continued drug pressure for an additional 2 years led to additional gene amplification (up to 12 copies) and the acquisition of *Pf*PNP catalytic site mutations (M183L and V181D) in separate clones. Of the 12-fold gene amplification, six copies of the amplified PNP gene were wild-type copies and with the other six copies corresponding to the mutant PNP (19). These amino acid mutations have not been previously reported for *Pf*PNP and appear to be novel point mutations in response to DADMe-ImmG drug pressure. The V181D clone exhibited decreased affinity for DADMe-ImmG but retained sufficient catalytic activity to provide the essential *Pf*PNP function (19). In contrast, the M183L mutant is severely compromised, displaying insufficient catalytic activity (a 17,000-fold decrease) to support the viability of the parasite (19). In this clone, the coexpressed native *Pf*PNP is fully susceptible to DADMe-ImmG inhibition. These parameters led to the hypothesis of hybrid native-M183L *Pf*PNP oligomers with altered properties that provide biological function.

Resistance against other antimalaria therapies is also known to develop *via* multiple mutational mechanisms. For example, *Pf*Kelch-13 mutations were reported to promote parasite survival through proteostatic regulation of other protein targets in artemisinin resistance and mutations and expression changes in *P. falciparum* P-glycoprotein homolog (*Pf*Pgh1) resulting in altered response to artemisin drugs (5, 20, 21). Mutations in *Pf*CRT along with polymorphisms in *P. falciparum* multidrug resistance gene 1 (*Pfmdr1*) have been reported to contribute to resistance to chloroquine and other quinine drugs (21, 22). As most clinical antimalaria drugs were designed based on phenotypic susceptibility and not to interfere with a single molecular target, several common mechanisms of drug resistance occur for multiple antimalarials (23, 24). An example is the increased copy number and mutations in *Pfmdr1* associated with resistance to quinoline and carbazole-based compounds and ACTs (24, 25, 26, 27).

Understanding the mechanism of drug resistance for a target-specific transition state analog informs therapeutic efficacy and potential measures to minimize resistance development. These include dosing schemes and multiple drug combinations to contain resistant strain development (28). For example, understanding the mechanisms of artemisinin resistance led to the recommendation of piperaquine (PPQ) as a partner drug with artemisinin in the treatment and prevention of malaria in children and pregnant women who fail to respond to the sulfadoxine–pyrimethamine combination (29, 30, 31). In regions with developing artemisinin resistance, triple combination therapies are advised by combining dihydroatermisinin–PPQ with mefloquine and artemether–lumifantrine with amodiaquine to combat the delay of parasite elimination, another hallmark of artemisinin resistance (28, 32).

Here we explore the compensatory mechanisms underlying the slow development of resistance to DADMe-ImmG. Low catalytic activity made it clear that the M183L mutation alone in PNP is incompatible with parasite survival. Genomic sequencing of the resistant parasites revealed that the M183L mutation was present in approximately one-half of the sequencing reads with the other half representing native *Pf*PNP copies. The crystal structure of *Pf*PNP shows a homohexamer with a trimer-of-dimer structure (Fig. 2; PDB ID: 1NW4). Because the point mutants are present in half of the sequences, we hypothesized that a hybrid expression of native and mutant subunits may be driving resistance. We engineered a fusion protein with native and M183L subunits covalently linked with a peptide linker (mutPNPfus). The enzymatic characterization of this hybrid protein revealed different kinetic properties from the parent protein and demonstrated that amplification of both native and M183L genes permits formation of hybrid *Pf*PNP oligomers to provide an unusual resistance mechanism against DADMe-ImmG. We discuss the probability of such mutations arising in malaria therapy.

**Figure 2:**
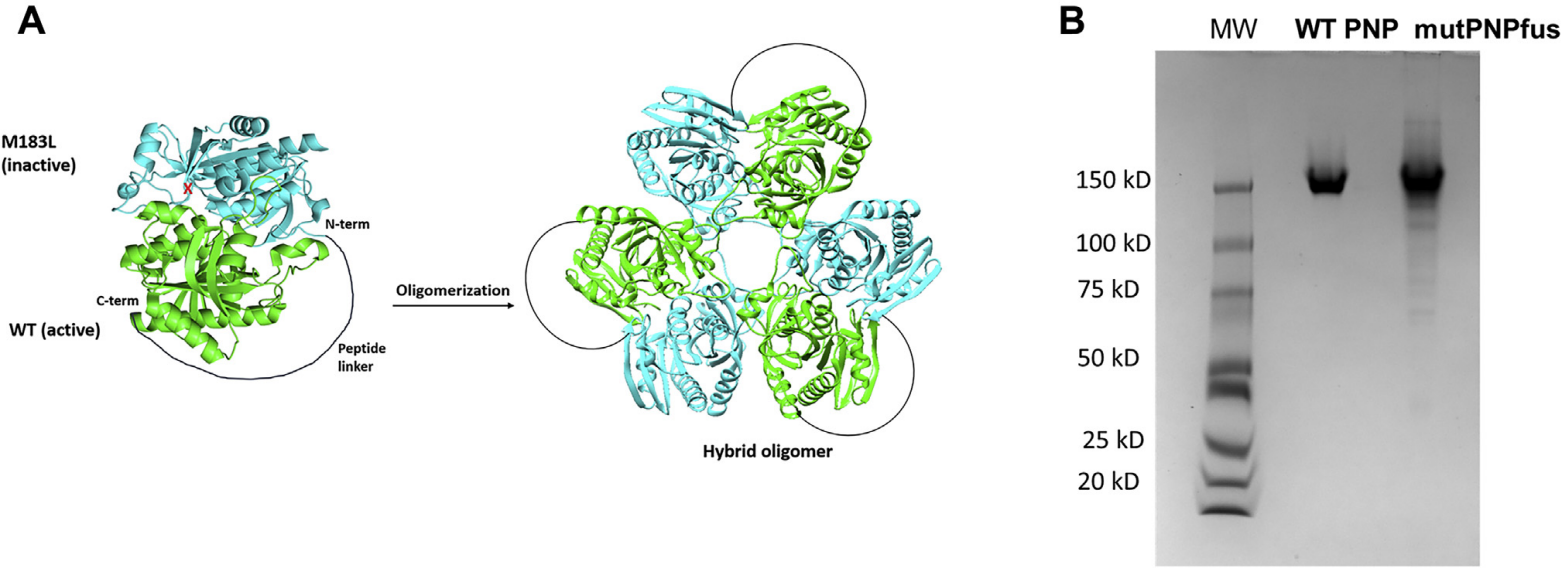
**Design of *Pf*PNPfus with covalently linked and alternating native and M183L subunits (mutPNPfus).** Expression in *E. coli* was used to form the fused hybrid dimer (*A*) followed by assembly of hybrid dimers to form the hybrid hexamer. natPNPfus has native PNP subunits fused with the same peptide linker. Native PAGE analysis for PNPs (*B*). Native *Pf*PNP (WT PNP) comigrates with mutPNPfus. The MW standards indicate both PNPs are hexameric.

## Results

### Covalent linking of wild-type and mutant subunits of *Pf*PNP

*Pf*PNP is a homohexamer with a trimer-of-dimer conformation (Fig. 2). The M183L mutation occurs between the catalytic site and the subunit interface, altering the geometry of the catalytic site to relocate Tyr160 into the catalytic site and thus interfere with both catalysis and the binding of DADMe-ImmG (Fig. 1). Crystallization studies with the M183L mutant yielded crystals without inhibitor in the catalytic site, consistent with the weak catalytic activity and weak inhibitor binding of M183L *Pf*PNP (19). In the DADMe-ImmG-resistant parasites expressing M183L *Pf*PNP, half the genomic sequences expressed native enzyme, therefore we hypothesized that resistance was achieved by formation of hybrid hexamers of *Pf*PNP containing combinations of native and mutant subunits. The trimer-of-dimer structure of *Pf*PNP enabled the design of covalently linked C-terminus of the native subunits to the N-terminus of the M183L subunits with a 20 amino acid peptide linker (ASGAGGSEGGGSEGGTSGAT) (mutPNPfus) for expression in *E. coli* (Fig. 2). The glycine and serine-rich peptide linker was selected as it is known to provide a flexible linker. In native *Pf*PNP, N and C termini are 50 Å apart and the extended length of the linker peptide is 70 Å. We proposed that this linked heterodimer would form a hybrid hexamer consisting of the cross-linked dimer unit with alternating native and M183L subunits. After expression and purification, we investigated the oligomer conformation of the fusion protein using nondenaturing gel electrophoresis, where the fusion protein migrated similar to native *Pf*PNP, establishing that native and hybrid fusion *Pf*PNPs have the same hexameric organization (Fig. 2).

### Kinetic parameters of fused PNPs (PNPfus)

The catalytic activity of M183L *Pf*PNP, where each subunit carries the M183L mutation, has been reported to be insufficient to support the essential function of *Pf*PNP *in vivo* (19). The catalytic properties of the mutPNPfus enzyme were characterized for comparison to native, all-M183L *Pf*PNP and cross-linked native *Pf*PNP (natPNPfus) using Michaelis–Menten kinetics. The natPNPfus construct provided a control for the kinetic parameters altered by the cross-linking. It was obtained by expressing a fusion protein of covalently linked native *Pf*PNP subunits with the same cross-linker sequence and also maintained hexameric oligomer structure (Fig. S1).

**Figure 3:**
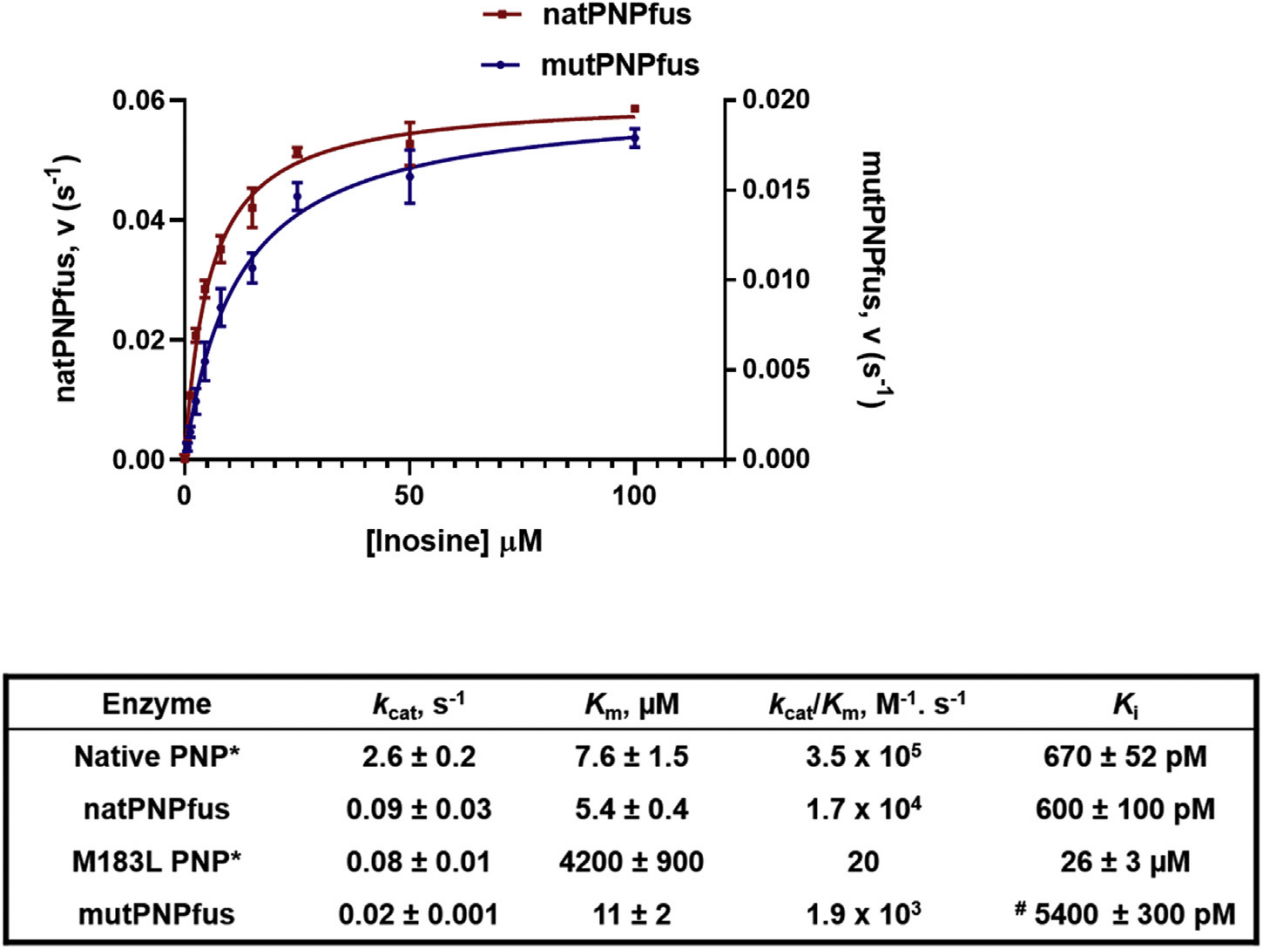
**Kinetic properties of PNP-fused subunit constructs.***Upper panel* shows fused native *Pf*PNP (*red*; natPNPfus) and and fused hybrid native-M183L subunits (*blue*; mutPNPfus). Substrate saturation curves show similar *K*_m_ but decreased *k*_cat_ for mutPNPfus. Kinetic constants for native *Pf*PNP and the fully mutant M183L *Pf*PNP are compared and summarized in the table. ∗ Previously reported (19). # *K*_i_ value determined from Equation 2. All other *K*_i_ values were determined from the equations for tight-binding inhibition (Equation 1; (35)).

The *K*_m_ values for native *Pf*PNP, natPNPfus, and mutPNPfus were similar at 7.6, 5.4, and 11 μM, respectively (Fig. 3). natPNPfus is catalytically slower than native *Pf*PNP, with *k*_cat_ values of 0.09 and 2.6 s^−1^, respectively. However, the natPNPfus construct serves as a control for the fusion construct carrying alternating native and M183L subunits (mutPNPfus). *Pf*PNP has a catalytic site functional group near the N-terminus, His7. It orients the 5'-hydroxyl group of the substrate to form an electrostatic interaction with the ribosyl 4’-hydroxyl group to facilitate formation of the ribocationic transition state (Fig. 1) (15).

Native *Pf*PNP has a robust *k*_cat_/*K*_m_ of 3.5 × 10^5^ M^−1^s^−1^. Fully mutated M183L *Pf*PNP is functionally inert relative to native *Pf*PNP, with the *K*_m_ value increased 780-fold, and the *k*_cat_ decreased 33-fold for a *k*_cat_/*K*_m_ of 20 M^−1^s^−1^, a 17,000-fold decrease in catalytic efficiency (19). The catalytic defect arises from the consequences of Tyr160 entering the catalytic site to hinder substrate entry (Fig. 1). This mutation also eliminates tight binding of the DADMe-ImmG transition state analog, increasing the dissociation constant from 670 pM in native *Pf*PNP to 26 μM in M183L *Pf*PNP, a factor of 39,000 (19). The mutation has made the subunit resistant to inhibition by DADMe-ImmG, but it has also rendered the enzyme to be catalytically weakened. When *Pf*PNP native and M183L subunits are coexpressed in resistant *P. falciparum* parasites, hybrids can form, exemplified here by mutPNPfus with alternating native and M183L subunits.

The structural control for the linked-subunit construct compares linked native *Pf*PNP (natPNPfus) with linked, alternating native *Pf*PNP and M183L subunits (mutPNPfus). In this comparison, the *k*_cat_ value for mutPNPfus is 4.5-fold slower than natPNPfus, and the catalytic efficiencies (*k*_cat_/*K*_m_) differ by a factor of 9. With 12-fold gene amplification (approximately equal expression of native and M183L sequences), the catalytic potential is similar to the parent parasite. Excess target expression is of limited biological value if the hybrid oligomers remain equally sensitive to DADMe-ImmG inhibition, therefore we quantitated the inhibitor action.

### Inhibition of mutPNPfus by DADMe-ImmG

**Figure 4:**
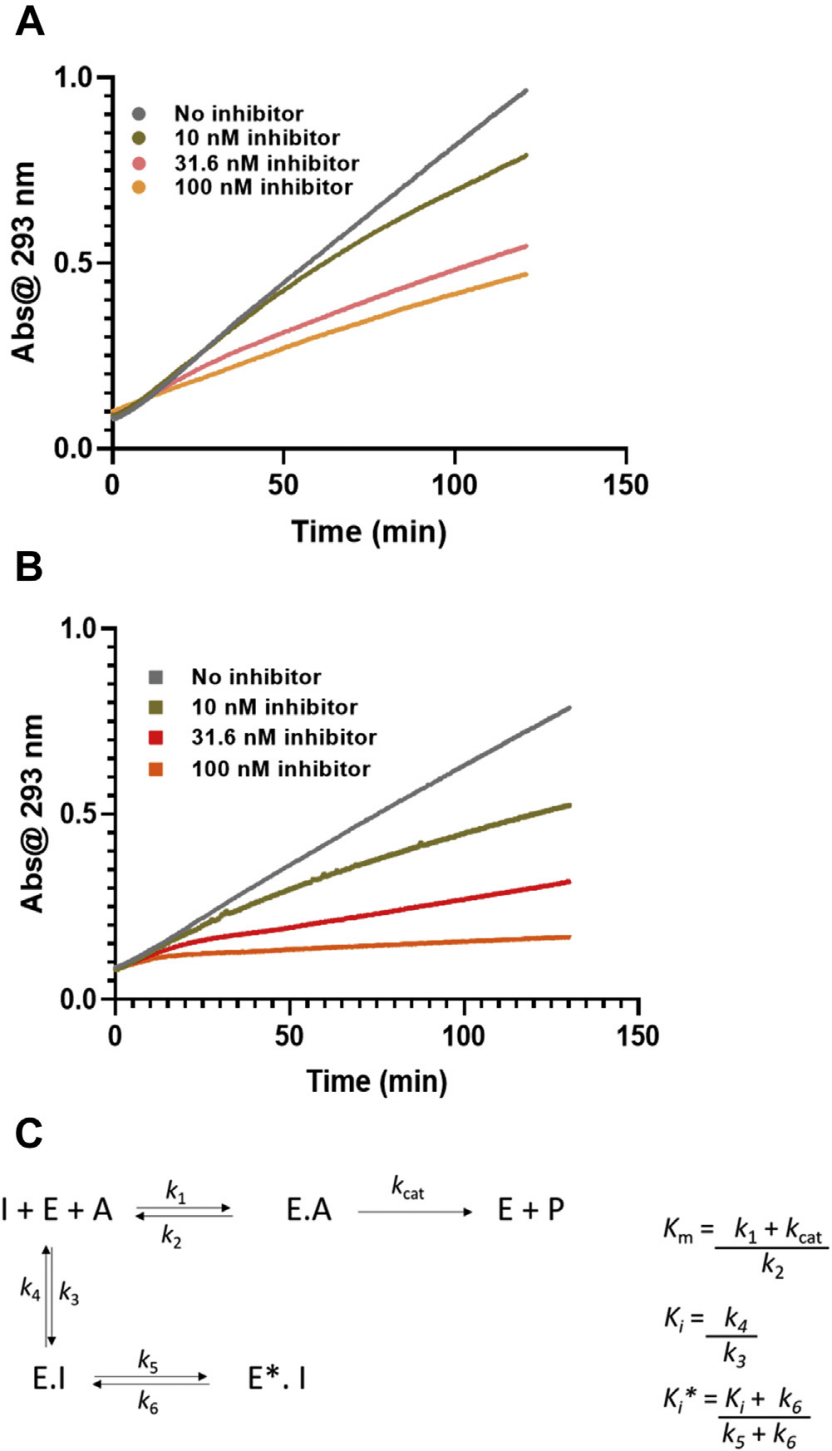
**Slow-onset inhibition of *Pf*PNPfus proteins with DADMe-ImmG.***A*, mutPNPfus and *B*, natPNPfus. In (*C*), the two-step process for slow-onset tight binding inhibition. Where I, E, A, and P are inhibitor, enzyme, substrate, and product, respectively.

DADMe-ImmG is a transition state analog of native *Pf*PNP and induces a slow-onset isomerization process leading to the formation of a tight-binding enzyme-inhibitor complex with a final dissociation constant of 670 pM (Fig. 4; (33, 34)). The slow-onset inhibition is experimentally observed in inhibition kinetics with a large excess of substrate that is replaced by the tight binding of transition state analog (Fig. 4). Inhibition curves of natPNPfus revealed a distinct two-phase inhibition (Fig. 4*B*), with near-complete inhibition following slow-onset at 100 nM DADMe-ImmG. In contrast, the mutPNPfus displayed a less pronounced slow-onset phase and weaker inhibition by DADMe-ImmG (Fig. 4*A*).

**Figure 5:**
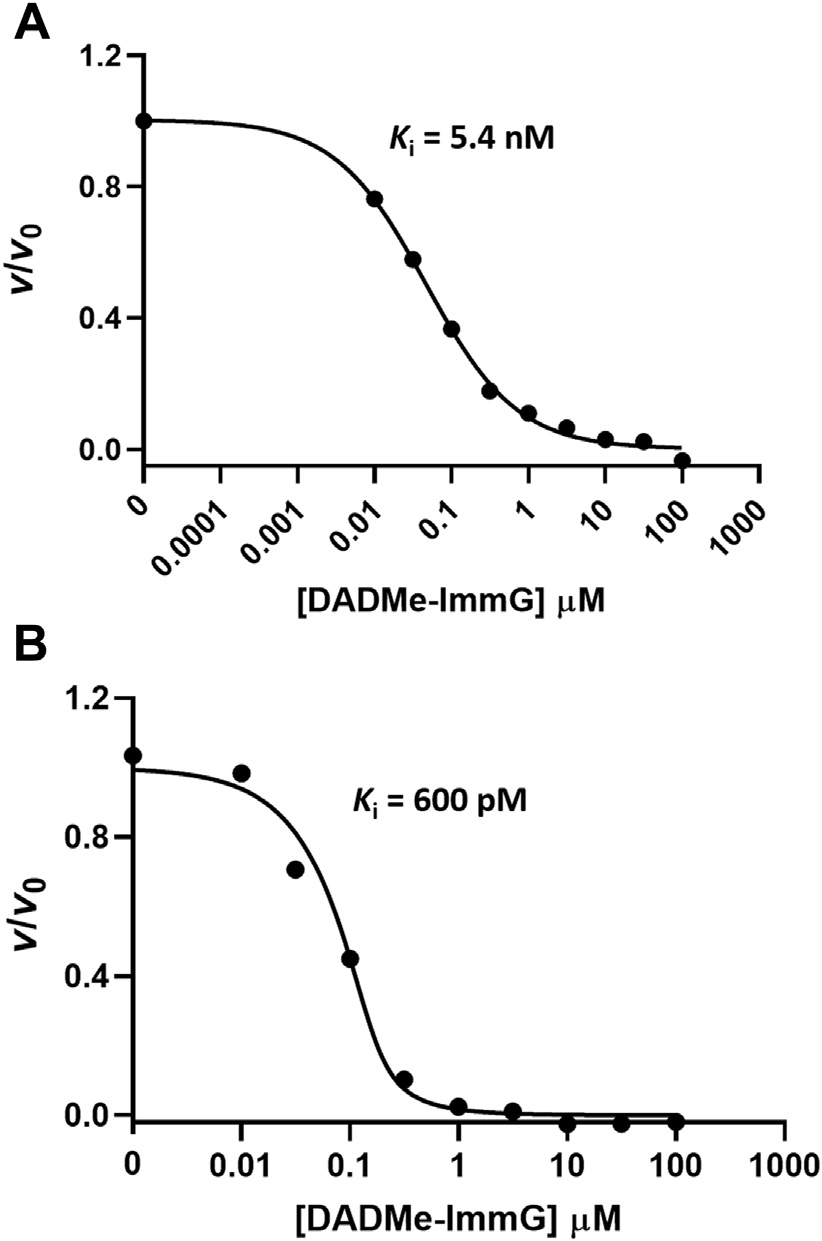
**DADMe-ImmG inhibition curves for *Pf*PNPfus.***A*, inhibition of mutPNPfus by DADMe-ImmG. The *K*_i_ was determined from Equation 2. *B*, inhibition of the natPNPfus control construct by DADMe-ImmG fit to the Equation 1 (35).

A feature of tight-binding inhibition assays is that inhibitor and enzyme concentrations are often similar and tight binding to the enzyme partitions a significant fraction of total inhibitor onto the enzyme, a characteristic included in the affinity calculations using the Morrison equation for tight-binding inhibitors (Equation 1) (35), where *v* and *v*_o_ are the initial velocities for determining *K*_i_ (*K*_i_ ∗ if slow-onset inhibition occurs), [E_o_] is the total enzyme concentration, and [I] is the inhibitor concentration. This approach was necessary for native *Pf*PNP and for natPNPfus. In contrast, mutPNPfus binds inhibitor less tightly and gave poor fits to the Morrison equation. Therefore, the *K*_i_ value was determined from the Cheng–Prusoff equation by first fitting the data to the classical IC_50_ equation (Equation 2; (36)), where S is the substrate concentration and *K*_m_ is the substrate concentration at the *k*_cat_/2 value. The mutPNPfus enzyme had a *K*_i_ value of 5.4 nM for DADMe-ImmG compared with a value of 600 pM for natPNPfus (similar to native *Pf*PNP; Fig. 5). The mutPNPfus enzyme displayed an 8.9-fold increase in the *K*_i_ for DADMe-ImmG, and this decreased inhibitor affinity is coupled to a similar decrease in catalytic efficiency (ninefold) when compared with the natPNPfus control construct (Fig. 3).

(1) $$v\;=\;vo\left(1-(\left({\left[E\right]}_{o}+\left[I\right]+{Ki}^{*}\right)-\sqrt{{\left({\left[E\right]}_{o}\;+\;\left[I\right]\;+\;{Ki}^{*}\right)}^{2}\;-\;4{\left[E\right]}_{o}\left[I\right]}\right)/(2{\left[E\right]}_{o}))$$

(2) $$K_{i}=\frac{IC_{50}}{1+\left(S/K_{m}\right)}$$

### Effect of peptide linker on catalytic activity

Concerned that the 20 amino acid linker was too short for optimal catalytic activity of the fused-subunit enzymes, we varied the length of the linker by increasing the peptide to 24 amino acids (ASGAGGSEG**SGSG**GGSEGGTSGAT). The construct containing the 24 amino acid peptide linker was expressed and characterized as described for the constructs with the 20 amino acid peptide linker. Kinetic properties with the 24 amino acid link were similar to those for PNPfus protein with the 20 amino acid linker (Figs. S3 and S4, Table S1). Therefore, increasing the linker length did not improve the catalytic properties of PNPfus.

A Tobacco Etch Virus (TEV) protease cleavage site was included into the N-terminal portion of the peptide linker and cleaved after the purification of PNPfus (natPNPfus and mutPNPfus). This cleavage freed the N-terminal region to provide freedom of motion to the His7 catalytic site function. Native PAGE gel analysis suggests that the TEV-cleaved constructs have similar hexameric organization as native *Pf*PNP (Fig. S5). Kinetic properties of constructs with cleaved peptide linkers indicated *k*_cat_ values increased tenfold for both mutPNPfus and natPNPfus relative to their uncleaved constructs, translating to an order of magnitude increase in the catalytic efficiency (*k*_cat_/*K*_m_) for mutPNPfus and a fivefold increase for natPNPfus. The *K*_m_ values for inosine and the *K*_i_ values for DADMe-ImmG remained unchanged for both mutPNPfus and natPNPfus indicating that the mobility of His7 is affected in the *Pf*PNPfus proteins to reduce the catalytic activity (Table 1). In all constructs, the decreases in catalytic activity and affinity for DADMe-ImmG are similar, in agreement with transition state theory that altered catalytic function reflects in the affinity of transition state analogs (19).

**Table 1:** Kinetic parameters of *Pf*PNPfus with cleaved peptide linker compared with native *Pf*PNP

Enzyme (cleaved linker peptide)	*k*_cat_, s^−1^	*K*_m_, μM	*k*_cat_/*K*_m_, M^−1^ s^−1^	*K*_i_
∗Native PNP	2.63 ± 0.15	7.6 ± 1.5	3.5 × 10^5^	670 ± 52 pM
natPNPfus	0.7 ± 0.03	7.9 ± 0.4	8.6 × 10^4^	745 ± 20 pM
mutPNPfus	0.14 ± 0.04	9.8 ± 2.5	1.4 × 10^4^	2100 ± 100 pM

An increase in catalytic function is observed for all fusion protein constructs.

∗ Previously reported (19).

### Catalytic site titration of PNPfus with DADMe-ImmG

**Figure 6:**
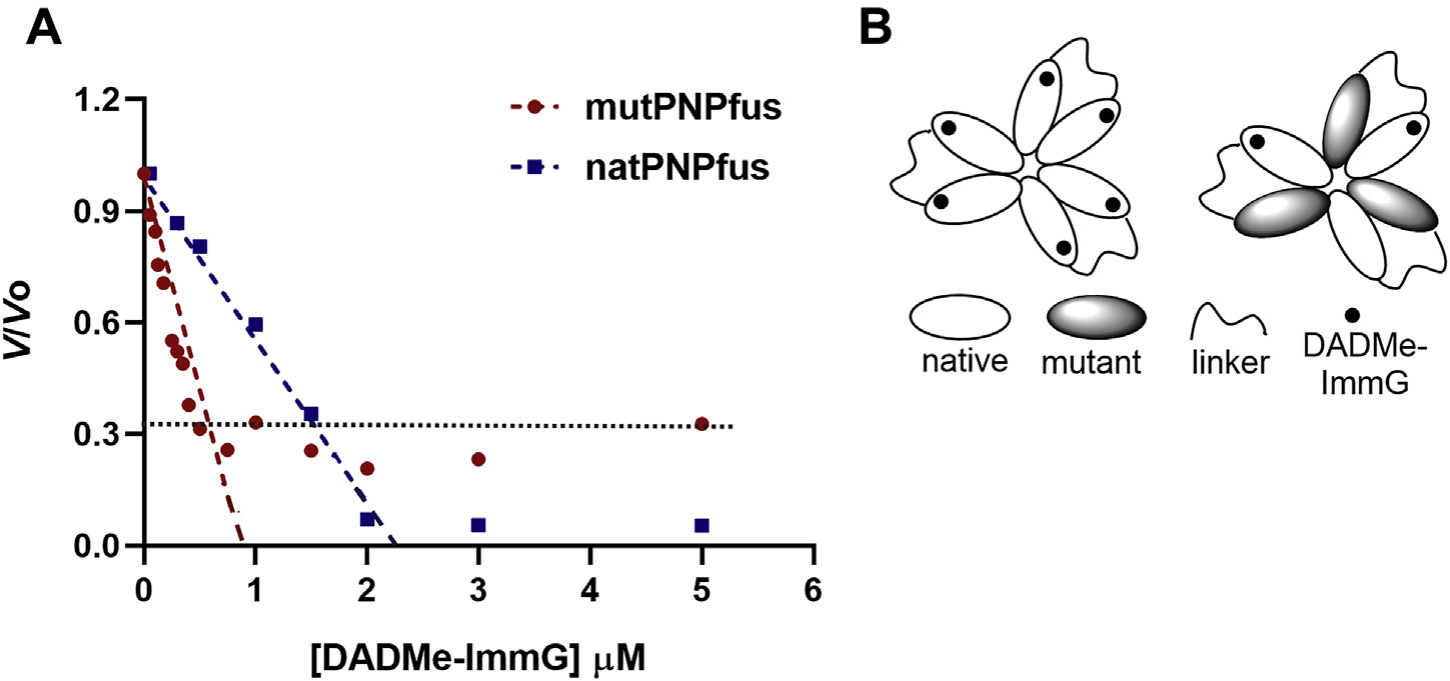
**Catalytic site titration of fused PNPs with the transition state analogue DADMe-ImmG.***A*, plots fractional activity (*V/Vo*) *versus* DADMe-ImmG concentration. Equal protein subunit concentrations (10 μM) of both mutPNPfus and natPNPfus were incubated with inhibitor. Abscissa intercepts for the titration at 1 and 2 μM, respectively. natPNPfus is fully inhibited by DADMe-ImmG. mutPNPfus has three functional catalytic sites and demonstrates 2/3 inhibition when titrated with DADMe-ImmG. *B*, a cartoon demonstrating the titration of DADMe-ImmG into the catalytic sites of mutPNPfus and natPNPfus.

Equal protein subunit concentrations (10 μM) of both mutPNPfus and natPNPfus were incubated with DADMe-ImmG to quantitate residual catalytic activity. As predicted, DADMe-ImmG binding to enzyme active sites was one-half of natPNPfus as in mutPNPfus. Abscissa intercepts for the titration at 1 μM and 2 μM, respectively, demonstrate that approximately 20% of the total enzyme protein is catalytically active (Fig. 6*A*). natPNPfus is fully inhibited by stoichiometric DADMe-ImmG titration, filling all six catalytic sites. mutPNPfus has three functionally catalytic sites and demonstrates 2/3 inhibition corresponding to binding to two out of three sites, suggesting negative cooperative inhibitor binding to the third site (Fig. 6).

## Discussion

Understanding mechanisms of drug resistance in *P. falciparum* is paramount to the selection of drug candidates to reduce development of drug resistance. Here, laboratory-cultured *P. falciparum* with resistance developed to the transition state analog, DADMe-ImmG, are characterized at the functional level of the protein target, *Pf*PNP. Three years of continuous drug pressure were required to generate robust drug resistance, supporting the hypothesis that mutations away from drug binding of a transition state analog are also mutations away from biological catalytic function (19, 37). Target gene amplification and target point mutations were both required to establish robust resistance, and here we demonstrate the unusual mechanism of mixed-subunit hybrid hexamers as the likely mechanism for resistance to DADMe-ImmG in *P. falciparum*.

### Covalently linked native and mutant subunits of *Pf*PNP

Covalent subunit peptide links can be used to examine the nature of multimeric proteins where functional and mutated catalytic site subunits are joined to recapitulate the multimeric effects. For example, we have used this approach to examine cooperativity in the catalytic sites of a bacterial methylthioadenosine nucleosidase (38). The 20 amino acid linker containing glycine and serine residues is conformationally flexible and was selected to permit a natural interface interaction between the subunits. Active and mutant subunits linked in this way formed active hexamer conformations, as established by migration in native electrophoresis. Thus, the combination of six subunits, molecular weight of approximately 26 kDa, recreated the hexameric protein with a molecular weight near 160 kDa (18).

### Transition state analog affinity reflects catalytic power

Transition state analogs have been proposed to be exceptional candidates for drug development, since they mimic the properties linked to catalysis at the instant of the transition state (19, 39). Thus, catalytic site mutations that decrease binding of the transition state analog are also proposed to reduce catalytic efficiency by a similar amount (37, 39). This relationship appears clearly in the enzymes and constructs described here. M183L *Pf*PNP has lost 17,000-fold catalytic efficiency with a similar decrease by a factor of 39,000 in DADMe-ImmG affinity. Likewise, comparison of natPNPfus with mutPNPfus demonstrates a ninefold difference in catalytic efficiency and 8.9-fold in DADMe-ImmG binding affinity. Development of this degree of resistance in parasite *Pf*PNP required 3 years of increased drug pressure in the cultured parasites adding to the evidence that tight-binding inhibitors have low resistance proclivities (19, 40). As typical therapy periods are days to weeks in malaria treatment, drug resistance to transition state analogs directed against *Pf*PNP has a low probability to generate resistance in the field.

### Kinetic parameters and DADMe-ImmG susceptibility of PNPfus

Kinetic characterization of the hybrid native-mutant *Pf*PNP protein revealed catalytically distinct properties from native *Pf*PNP protein and explains the survival of resistant parasites. The mutPNPfus protein had decreased catalytic efficiency from the wild-type constructs, independent of the length of the peptide linker or its cleavage formation of the hybrid hexamers. The decreased catalytic function due to the presence of a covalent N-terminal to C-terminal linker is attributed to a slower His7 catalytic function at the active sites of the linked constructs. Thus, constructs in which the peptide linker has been cleaved show increased catalytic function (Table 1). However, results from catalytic site titration experiments revealed that approximately 20% of the total enzyme protein was catalytically active (Fig. 6). This result, when taken into consideration for *k*_cat_ values determined for the TEV cleavage constructs of mutPNPfus and natPNPfus, means that turnover number and catalytic efficiency for natPNPfus are restored to native PNP levels when the linker is cleaved and the *k*_cat_ for mutPNPfus is corrected for active enzyme concentration. This translates to a sixfold decrease in catalytic efficiency for mutPNPfus when compared with natPNPfus. In any of the constructs, the mutPNPfus exhibits decreased catalytic efficiency, due to the presence of inactive M183L subunits in the complex but possibly also due to the intersubunit contacts between the inactive and native (active) subunits.

Hypoxanthine salvage is essential for the purine auxotrophic metabolism of *P. falciparum* parasites, requiring that parasites expressing the combination of native and M183L *Pf*PNP constructs be functional in *Pf*PNP activity, even with the presence of elevated DADMe-ImmG. Purine nucleoside phosphorylase activity in the resistant organisms results from a combination of target overexpression from gene amplification and from a reduced affinity of the hybrid construct for DADMe-ImmG.

It is noteworthy that the degree of loss of catalytic efficiency is equal to the loss in affinity for the inhibitor. This is a feature of transition state analogs that incorporate the intrinsic catalytic properties of the enzyme. Considering all comparisons of the natPNPfus and the mutPNPfus constructs, the affinity for DADMe-ImmG is decreased in every mixed-subunit hybrid, from three- to nine-fold (average sixfold). While the catalytic function (*k*_cat_/*K*_m_) is also decreased by sixfold in the most active of the mutPNPfus constructs, the 12-fold gene amplification for *Pf*PNP in the resistant strains provides twofold excess compensation for the decreased catalytic efficiency (Table 1). The result is robust resistance compared with the native parasites (13). It should be noted that the alternating (active-inactive)_3_ construct described here is only one of the possible hexameric arrangements when approximately equal amounts of native and mutant subunits are expressed. Others may have distinct kinetic properties, but are not as experimentally accessible. For the *Pf*PNP target, the results indicate that increased cellular expression of native and mutant subunits forms hybrids that exhibit reduced affinity for the transition state analog DADMe-ImmG and compensate for the reduced catalytic function with the overproduction of protein. Increased protein expression also increases the target size for saturation by the transition state analog. The slow generation of resistance to DADMe-ImmG in *P. falciparum* culture is due to the requirement for multiple genetic events. This mechanism argues for the slow development of drug resistance to transition state analogs of *Pf*PNP in the field.

## Conclusions

Drug pressure has led to the development of resistance in *P. falciparum* to every therapeutic agent in common use. Rapid spread of resistance to single agents has led to the current WHO recommendation that all antimalarial therapy be conducted by drug combinations. Even then, resistance is developing. New agents, slow to induce resistance, are a continuing need. Transition state analogs bind tightly to their targets, giving extended biological action *in vivo* by displaying prolonged inhibition of their target. Mutation away from binding of transition state analogs is also mutation away from biological function of the target enzyme. Drug resistance against DADMe-ImmG is achieved *via* target overexpression combined with active site mutations that form hybrid *Pf*PNP oligomers with decreased affinity for the inhibitor. The essential function of PNP in hypoxanthine salvage for *P. falciparum* drives the production of an unusual hybrid protein of active and inactive subunits. This resistance mechanism coupled with the slow development of drug resistance to DADMe-ImmG highlights its potential for use in antimalaria combination therapies.

## Experimental procedures

### Materials

DADMe-ImmG was a generous gift from Dr Peter Tyler of the Ferrier Research Institute, New Zealand. BugBuster was purchased from EMD Millipore. Electrophoresis gels and buffers were purchased from Bio-Rad Laboratories. All buffers and media were purchased from Sigma-Aldrich and Fisher Scientific, respectively. Xanthine oxidase was purchased from Sigma-Aldrich.

### Expression and purification of *Pf*PNPfus

The gene sequence for wild-type *P. falciparum* PNP (*Pf*PNP) was linked to the gene sequence for M183L mutant *Pf*PNP *via* a 20 amino acid peptide linker. The gene sequence was codon optimized and cloned into the pET28 a (+)- TEV plasmid containing the kanamycin resistant gene (GenScript). An N-terminal 6x histidine tag was added for affinity purification of the protein. The plasmid-containing BL21 DE3 *E. coli* cells were grown at 37 °C to an OD_600 nm_ of 0.4 to 0.6 and expression was induced with 1 mM IPTG overnight at 18 °C. Cells from 8 L of culture were disrupted using BugBuster cell lysis reagent at room temperature, and the soluble portion was harvested after centrifugation at 13,000*g* for 20 min. The supernatant was mixed with 15 mL Ni-NTA preequilibrated with 50 mM Tris-HCl pH 8, 200 mM NaCl, 5 mM imidazole, and 1 mM DTT and incubated for 45 min at 4 °C with shaking. An elution column was formed from this mixture and washed with 150 mL of buffer containing 50 mM Tris-HCl pH 8, 200 mM NaCl, 5 mM imidazole, and 1 mM DTT. *Pf*PNPfus was eluted from the Ni-NTA column by gravity flow with 25 mL of buffer containing 50 mM Tris-HCl pH 8, 200 mM NaCl, 1 mM DTT with a gradient imidazole concentration of 50 to 500 mM. The protein was dialyzed against buffer containing 50 mM phosphate pH 7.6 and 1 mM DTT with three buffer exchanges. Protein purification and dialysis were at 4 °C. The enzyme was concentrated to approximately 1 mL and the concentration of *Pf*PNPfus was determined by absorption at 280 nm with the extinction coefficient determined from the ProtParam server. The purified protein was aliquoted, frozen in liquid nitrogen, and stored in −80 °C. natPNPfus and mutPNPfus were stored at concentrations of 160 μM and 360 μM respectively.

### Mutagenesis and cloning

Covalently linked native dimers of *Pf*PNP (natPNPfus) were generated by site-directed mutagenesis of the *Pf*PNP native-M183L (mutPNPfus) plasmid, using the QuikChange lightning site-directed mutagenesis kit (Agilent Technologies, California USA) and the primer pair (Primer 1: GCGGCGGTTGTTGAGATGGAATTAGCTACCC and primer 2: GGGTAGCTAATTCCATCTCAACAACCGCCGC) designed on the QuikChange primer design tool. Site-directed mutagenesis was performed according to the manufacturer’s protocol. Plasmid sequencing (GENEWIZ) was done to confirm the correct sequence of the natPNPfus plasmid. PNPfus constructs containing 24 amino acid peptide linker (ASGAGGSEGSGSGGGSEGGTSGAT) and the constructs containing the TEV cleavage site in the N-terminus of the linker were designed and were produced by GenScript.

### Enzyme assays

The catalytic activity for PNPfus proteins was determined in continuous assays by coupling the reaction of PNP to that of xanthine oxidase and measuring the absorbance of uric acid formation at 293 nm. Briefly, the concentration of inosine was varied from 0 to 100 μM in 50 mM phosphate buffer at pH 7.4, and the formation of hypoxanthine was coupled to the formation of uric acid monitored by the the absorption increase at 293 nm and 25 °C. Reactions were initiated by addition of 50 mU xanthine oxidase and 500 nM mutPNPfus or 100 nM natPNPfus.The *K*_m_ and the *k*_cat_ values were determined by fitting to the Michaelis–Menten equation.

### Inhibition assays

Kinetic assays of PNPfus for inhibitor susceptibility were performed at 100 μM inosine and varying the concentration of DADMe-ImmG from 0 to 100 μM in 50 mM phosphate buffer, pH 7.4 at 25 °C. The formation of hypoxanthine *via* the xanthine oxidase coupled assay was monitored by the absorption increase of uric acid at 293 nm. In total, 50 mU xanthine oxidase and 500 nM mutPNPfus or 100 nM natPNPfus were added to initiate the reaction. The *K*_i_ values were determined by fitting the data to the Morrison equation for natPNPfus using the Prism 8 matrix. The *K*_i_ for mutPNPfus was determined from the Cheng–Prusoff equation by first fitting the data to the four-parameter IC_50_ equation in Prism 8.

### Catalytic site titration

mutPNPfus and natPNPfus were each incubated with stoichiometric concentrations of DADMe-ImmG for 30 min at 25 °C. A 100-fold dilution was made into an assay mix containing 500 μM inosine and 50 mM phosphate buffer pH 7.4. The reaction rates were determined from monitoring the increase in uric acid absorbance at 293 nm using the xanthine oxidase coupled assay. Data was analyzed in Prism 8 using segmental linear regression.

## Data availability

All data that support the findings of this study are contained within the article and its supporting information.

## Conflict of interest

The authors declare that they have no conflicts of interest with the contents of this article.
