# Supplementary material for: A resistant mutant of Plasmodium falciparum purine nucleoside phosphorylase uses wild-type neighbors to maintain parasite survival
Source: J Biol Chem. 2021 Jan 30;296:100342. doi: 10.1016/j.jbc.2021.100342 (PMC7949152; doi:10.1016/j.jbc.2021.100342)
Supplement: Supplemental Figures S1–S5 and Table S1 [file mmc1.pdf]

SUPPORTING INFORMATION

A resistant mutant of *Plasmodium falciparum* purine nucleoside phosphorylase uses wild-type neighbors to maintain parasite survival

Yacoba V.T. Minnow, Rajesh K. Harijan, Vern L. Schramm*

Department of Biochemistry, Albert Einstein College of Medicine, Bronx, New York 10461

*Corresponding Author:

Vern L. Schramm,

Department of Biochemistry, Albert Einstein College of Medicine,

1300 Morris Park Ave., Bronx, New York 10461

Telephone: 718-430-2813

Email: vern.schramm@einsteinmed.org

SUPPORTING INFORMATION

Oligomeric state of PNPfus

Purified PNPfus was resolved on a Superdex (16/60) size exclusion column into its oligomeric species. The activity of the different fractions was determined using the spectrophotometric assay outlined in the experimental procedures. Similar activity of the hexameric fraction and the higher-order oligomeric fraction was observed for natPNPfus suggesting the formation of the active hexameric enzyme in the presence of substrates or inhibitor.

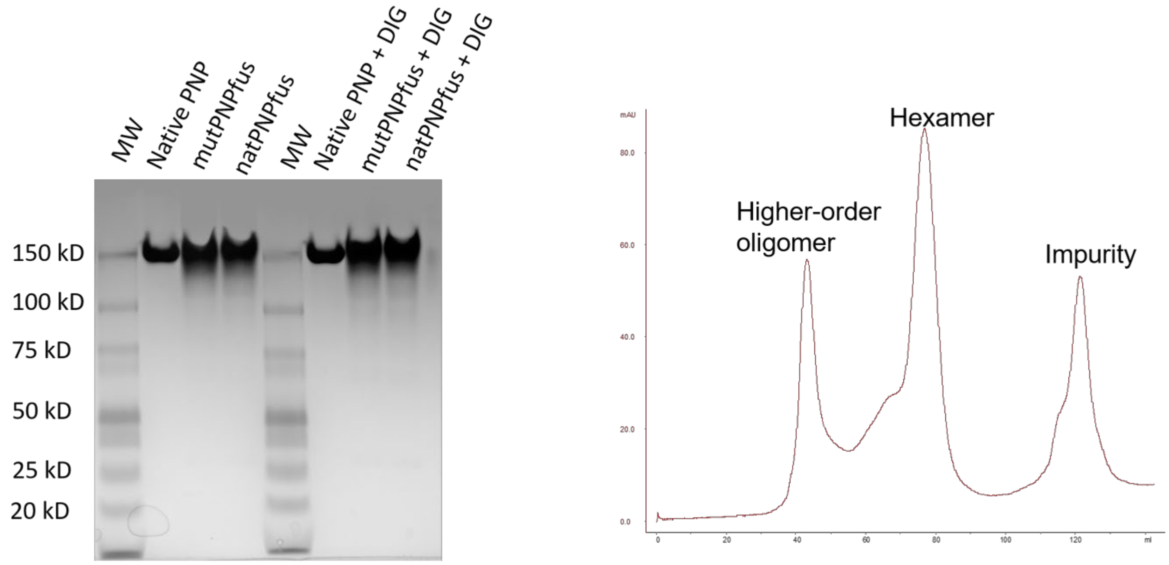

Figure S1. Native PAGE analysis for PNPs showing the comigration of native *Pf*PNP with both mutPNPfus and natPNPfus. DIG = DADMe-ImmG (left). Size exclusion of *Pf*PNPfus on FPLC showing different oligomeric fractions (right). These indicate that the engineered PNPs are hexameric.

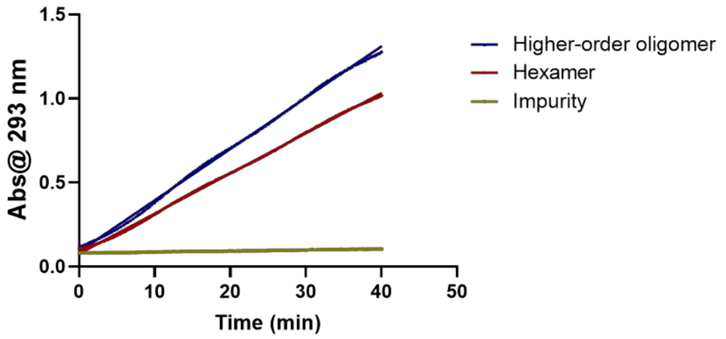

Fraction	rate (abs/min)
Higher-order oligomer	0.031
Hexamer	0.024
Impurity	0.0006

Figure S2. Activity of different fractions of natPNPfus from size exclusion. The hexameric fraction and higher-order oligomeric fraction show similar rates of activity.

Increasing peptide linker length of PNPfus

The kinetic constants of mutPNPfus and natPNPfus suggested an effect of the 20 amino acid linker on the rate of the enzyme. We investigated this by increasing the length of the peptide linker to 24 amino acids (ASGAGGSEGSGSGGGSEGGTSGAT). Protein purification and kinetic characterization were performed as outlined in the experimental procedures. The results were similar to that obtained with the mutPNPfus and natPNPfus containing the 20 amino acid peptide linker (Fig.3 and Table S1).

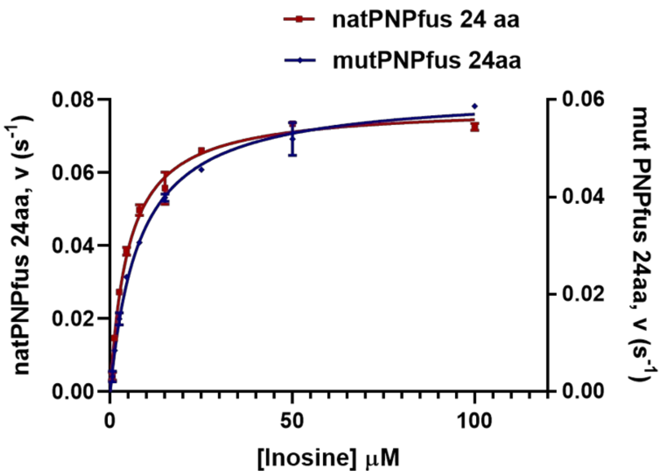

Figure S3. Steady state kinetics of *Pf*PNPfus with 24 amino acid linker (24 aa). The increased linker length did not substantially alter the enzymatic rates. Kinetic values summarized in Table S1.

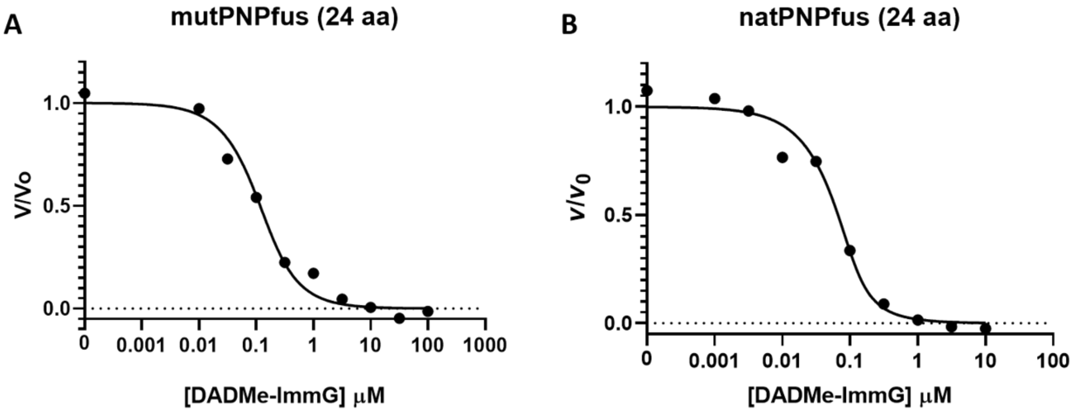

Figure S4. Inhibition of *Pf*PNPfus with 24 amino acid linker by DADMe-ImmG. Data were fit to the Morrison equation for the determination of K_i (main text equation 1). K_i values summarized in Table S1.

Table S1: Kinetic parameters of 24 amino acid linker *Pf*PNPfus compared to wild-type *Pf*PNP. The catalytic parameters are similar to the fusion enzyme with 20 amino acid linker.

Enzyme (24 amino acid linker)	k_{cat}, s^{-1}	K_m, μM	k_{cat}/K_m, M^{-1}. s^{-1}	K_i
*Native PNP	2.63 ± 0.15	7.6 ± 1.5	3.5×10^5	670 ± 52 pM
natPNPfus	0.08 ± 0.02	4.9 ± 0.5	1.6×10^4	780 ± 100 pM
mutPNPfus	0.06 ± 0.001	8.0 ± 0.7	7.7×10^3	5900 ± 300 pM

*Previously reported (see main text Fig. 3 and Table 1).

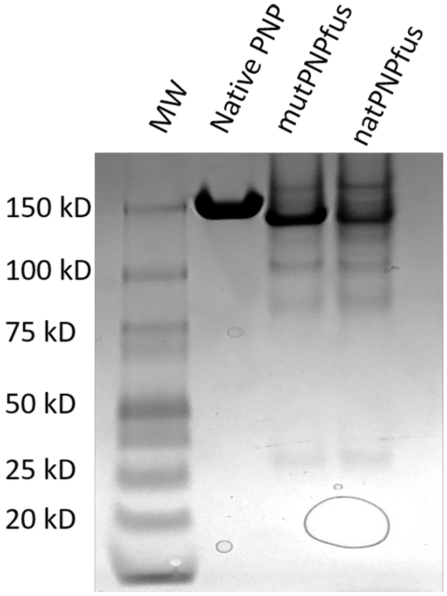

Figure S5. Native PAGE analysis for PNPs showing the comigration of native *Pf*PNP with both cleaved mutPNPfus and natPNPfus.
